# Beneficial and detrimental fungi within the culturable mycobiome of the Red Sea coral *Stylophora pistillata*

**DOI:** 10.1093/ismejo/wraf090

**Published:** 2025-05-03

**Authors:** Lior Granit, Rotem Levi, Nofar Lifshitz, Guilhem Banc-Prandi, Einat Zelinger, Britt Ronen, Judith Kraut-Cohen, Ankur Naqib, Stefan J Green, Maoz Fine, Oded Yarden

**Affiliations:** Department of Plant Pathology and Microbiology, The Robert H. Smith Faculty of Agriculture Food and Environment, The Hebrew University of Jerusalem, Rehovot 7610001, Israel; The Interuniversity Institute for Marine Sciences, Eilat 8810302, Israel; Department of Plant Pathology and Microbiology, The Robert H. Smith Faculty of Agriculture Food and Environment, The Hebrew University of Jerusalem, Rehovot 7610001, Israel; The Interuniversity Institute for Marine Sciences, Eilat 8810302, Israel; Department of Plant Pathology and Microbiology, The Robert H. Smith Faculty of Agriculture Food and Environment, The Hebrew University of Jerusalem, Rehovot 7610001, Israel; The Interuniversity Institute for Marine Sciences, Eilat 8810302, Israel; Laboratory for Biological Geochemistry, School of Architecture Civil and Environmental Engineering, École Polytechnique Fédérale de Lausanne (EPFL), 1015 Lausanne, Switzerland; Centre for Scientific Imaging, The Robert H. Smith Faculty of Agriculture, Food and Environment, The Hebrew University of Jerusalem, Rehovot 7610001, Israel; The Interuniversity Institute for Marine Sciences, Eilat 8810302, Israel; Department of Ecology, Evolution and Behavior, The Alexander Silberman Institute of Life Sciences, The Hebrew University of Jerusalem, Jerusalem 9190401, Israel; Institute of Soil, Water and Environmental Sciences, Agricultural Research Organization, Volcani Center, Rishon LeZion 7505101, Israel; Rush Research Bioinformatics Core Facility, Rush University, Chicago, IL 60612, United States; Genomics and Microbiome Core Facility, Rush University, Chicago, IL 60642, United States; The Interuniversity Institute for Marine Sciences, Eilat 8810302, Israel; Department of Ecology, Evolution and Behavior, The Alexander Silberman Institute of Life Sciences, The Hebrew University of Jerusalem, Jerusalem 9190401, Israel; Department of Plant Pathology and Microbiology, The Robert H. Smith Faculty of Agriculture Food and Environment, The Hebrew University of Jerusalem, Rehovot 7610001, Israel; The Interuniversity Institute for Marine Sciences, Eilat 8810302, Israel

**Keywords:** Stylophora, coral mycobiome, marine fungi, *Cladosporium*, *Stachybotrys*, ocean warming

## Abstract

The presence of fungi in the coral microbiome is increasingly recognized, yet their potential impact on the holobiont’s health, particularly under stress conditions, remains underexplored. To address this gap, we isolated over 200 strains (predominantly Ascomycota) from the common scleractinian Red Sea coral, *Stylophora pistillata*. Using conidia from a rare (*Stachybotrys chlorohalonata*) and a common (*Cladosporium halotolerans*) fungal symbiont, we investigated their effects on coral fragments maintained at ambient (25°C) and elevated (33°C) sea temperatures. Inoculation with *S. chlorohalonata* resulted in significant tissue loss, across both water temperature treatments. Conversely, inoculation with *C. halotolerans* did not result in visible effects at ambient temperature, but mitigated tissue loss at elevated temperature. This protective effect was accompanied by reduced expression of stress-induced peroxiredoxin-6 and Rad51 host genes, yet not that of Hsp70. Additionally, potential algal symbiont photosynthetic efficiency was higher by over 25% in the elevated temperature treatment, concurrent with higher bacterial diversity, including a marked reduction (>3-fold) in the proliferation of Vibrionaceae in the *C. halotolerans*-treated coral nubbins. These findings reveal the contrasting impacts of fungal symbionts on coral health, highlighting the dual roles of the mycobiome in influencing holobiont resilience under environmental stress.

Fungal symbionts are increasingly recognized as common members of reef-building coral microbiomes [1]. Coral–microbe interactions are known to affect coral physiology and health [2–4]. However, the diversity, functionality, and ecological roles of fungi within the coral holobiont remain poorly understood [5]. A prime example is *Aspergillus sydowii*, a fungal endophyte implicated in aspergillosis in Caribbean Sea fans [6, 7], whose pathogenicity appears to correlate with rising water temperatures [8].

Given the frequent association of fungi with corals, we hypothesized that shifts in their diversity or abundance could influence coral well-being, especially in a changing environment. To test this, we sampled the culturable mycobiome of *S. pistillata*, a well-studied common scleractinian coral in the Gulf of Aqaba, northern Red Sea [9]. We isolated a total of 207 purified strains, representing 26 families spread across three phyla, including two new taxa (described elsewhere) from *S. pistillata* (Fig. 1; Supplementary Tables S1 and S2). Our isolation procedure has known limitations [10] and it is likely that a significant number of additional taxa are associated with the holobiont. From this diverse collection, we selected two representatives for assessing their effects on the well-being of *S. pistillata* under thermal stress: (i) *S. chlorohalonata*, a rarely (<1%) found species in the coral, yet whose terrestrial counterparts are known for the production of metabolites who are toxic to other organisms [11] and (ii) *Cladosporium halotolerans*, a proven halotolerant species [12] which was one of the most common (~10%) species in our collection and also found in geographically adjacent samples of *Acropora loripes* [13].

**Figure 1:**
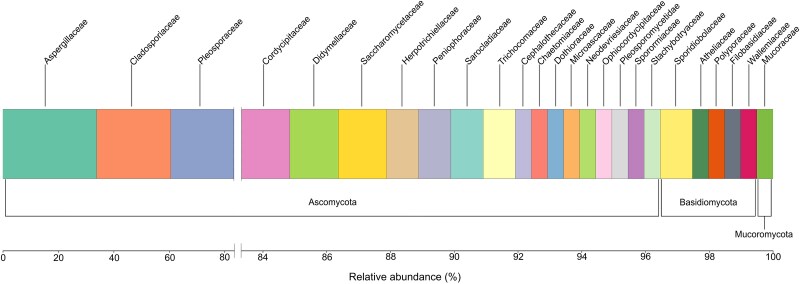
Relative abundance of cultured members of fungal families, grouped by phylum, isolated from *Stylophora pistillata* based on internal transcribed spacer sequences. The break in the *x*-axis between 80% and 84% was introduced to improve visualization of the less abundant taxa.

After a 48-h period of exposure to conidia of the two fungi at ambient sea temperature (25°C), the coral nubbins were washed with filtered sea water and transferred, along with uninoculated controls, to an open aquaria system maintained at either ambient or elevated (33°C) temperatures (Supplementary Fig. S1). *Stachybotrys chlorohalonata* induced rapid tissue loss, within 24 h post-inoculation, in the ambient temperature treatment (Fig. 2a and Supplementary Fig. S2), consistent with its known production of toxic metabolites [11], and was consequently excluded from further experiments. In contrast, *C. halotolerans* was determined, by microscopy, to adhere to the coral tissue and did not confer visible detrimental effects on the coral at ambient (25°C) temperature (Fig. 2a and Supplementary Fig. S3). Furthermore, it appeared to mitigate the detrimental effects, including tissue loss, observed at the elevated (33°C) temperature. Reduced tissue loss and a significant reduction in the expression of the coral host stress marker peroxiredoxin-6 ([14]; Fig. 2b) suggest mitigation of thermal stress. A similar, though statistically non-significant, trend was observed in the expression of another stress marker—Rad51. In contrast, the increase in Hsp70 expression in the heat-exposed coral was not significantly reduced in fungal-inoculated nubbins at the elevated temperature (Fig. 2b). This could be indicative of some host stress imposed by the fungus. Alternatively, this could be due to a differential effect on the host in which expression of Hsp70 was not hampered (as has been shown to occur in heat-resilient coral; [15]) by the presence of the apparently beneficial fungus. Coral inoculated with *C. halotolerans* exhibited higher photosynthetic symbiont photosystem II (PSII) activity at both ambient and elevated temperature conditions, as measured by pulse amplitude modulated fluorometer analysis (Fig. 2c). This corroborated the positive effect of the presence of *C. halotolerans*, demonstrating a measurable positive impact on the symbiotic dinoflagellates, known to be highly sensitive to a rise in sea temperature [16].

**Figure 2:**
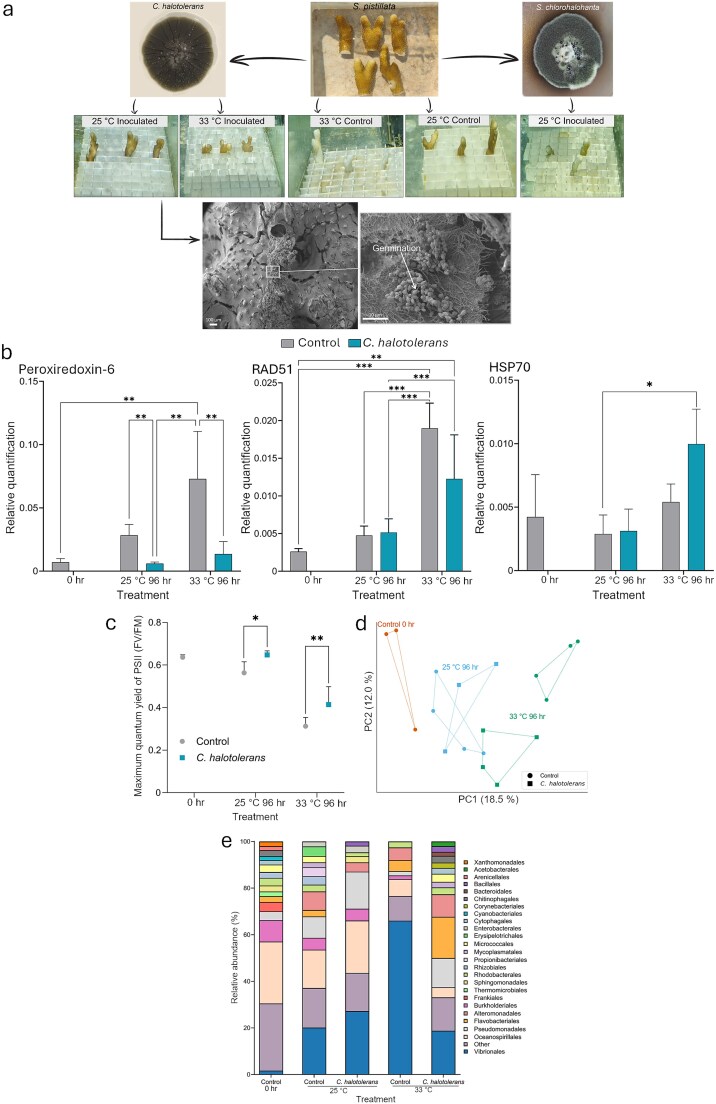
Effect of *Cladosporium halotolerans* and *Stachybotrys chlorohalonata* on *Stylophora pistillata* under thermal stress: (a) Nubbins of *S. pistillata* were inoculated with conidia of *S. chlorohalonata* or *C. halotolerans* (top row; for details, see experimental design fig. S1). Maintaining the nubbins at 25°C or 33°C resulted in adverse effects, ranging from tissue loss in nubbins at the high temperature and in the *S. chlorohalonata*-inoculated coral (regardless of temperature) to reduced tissue loss in nubbins inoculated with *C. halotolerans* (middle row). *C. halotolerans* conidia adhered and germinated on the coral surface (bottom row). Scanning electron micrograph (SEM) images of *S. pistillata* inoculated with *C. halotolerans*. The left panel (×60 magnification) shows an overview of the coral surface with fungal colonization, while the right panel (×2000 magnification) highlights conidia germination. SEM images were captured using a JEOL microscope (Vacc = 2.00 kV, detector = LED, WD = 10.0 mm). Scale bars: left panel = 100 μm, right panel = 10 μm. (b) Relative expression of three stress-related genes in *S. pistillata* as affected by temperature and inoculation with *C. halotolerans.* Statistical analysis was performed at each time point using two-way ANOVA, Tukey HSD post hoc test (* ≤ .05, ** ≤ .01 *** ≤ .001, *n* = 4 for peroxiredoxin-6 and RAD51, *n* = 3 for HSP70). Error bars represent standard deviation. (c) Mean maximum quantum yield of PSII (Fv/Fm) in *S. pistillata* coral fragments as affected by temperature and inoculation with *C. halotolerans.* Statistical analysis was performed at each time point using two-way ANOVA, Tukey HSD post hoc test (* < 0.05, ** < 0.01, *n* = 5). Comparisons represent differences between treatments within each temperature. Error bars represent standard deviation. (d) Principal coordinate analysis of microbial community composition based on Bray–Curtis distance matrices, visualizing differences of bacterial community structure between treatments and time points. The ordination illustrates the clustering of samples according to their microbiome profiles under the different temperature/fungal inoculation regimes. Results are based on *n* = 3 or *n* = 4 biological replicates per treatment. (e) Relative abundance of microbial communities at the order level across treatments. Orders representing <1.5% of the total abundance, or those without taxonomic classification at the order level, are grouped as “other.” Excluding the 0 h control, all samples were collected after 96 h.

Exposure to heat, *C. halotolerans* inoculation, and their combination affected the beta diversity of the bacterial community (Fig. 2d and Supplementary Table S4). Principal coordinate analysis based on Bray–Curtis dissimilarity showed distinct clustering between heat-stressed corals (33°C 96 h) with and without *C. halotolerans*, indicating fungal-driven shifts in bacterial composition under heat stress (Fig. 2d). A shift in alpha diversity revealed the occurrence of changes in bacterial richness across treatments (Supplementary Table S3 and Supplementary Fig. S5). The most notable differences were between the freshly harvested, 0 h, control, and nubbins incubated at 33°C, where the Shannon Index dropped from baseline levels of 5.12 to 3.09, indicating, as has been previously shown [17], that elevated temperatures negatively impact bacterial diversity. Inoculation with *C. halotolerans* also affected bacterial diversity at ambient temperature (Shannon index = 3.97), where it was lower than the control (4.78), yet at the elevated temperature, the presence of *C. halotolerans* resulted in higher (albeit not statistically significant within this restricted permitted coral sample size) bacterial diversity (Shannon index = 4.02) compared to the heat-stressed control (Supplementary Table S3 and Supplementary Fig. S5). Amplicon sequence variants related to the bacterial order Vibrionales [Proteobacteria (Supplementary Fig. S4)], whose increased abundance has been correlated with detrimental effects on the host [18, 19], were found to have higher relative abundance throughout the experiment, peaking in the heat-exposed control nubbins. However, this increase was markedly lower in nubbins inoculated with the fungus relative to the control, with the relative abundance of Vibrionales increasing by ~70% in heat-exposed control nubbins and only about 30% in heat-exposed inoculated nubbins (Fig. 2e and Supplementary Table S4). Our findings highlight the dual nature of the influence of fungal presence within the coral holobiont, at least under the conditions tested in this study.

High fungal diversity may be linked to bleaching susceptibility [20], yet our results indicate that even increasing the abundance of a single fungal species may have the ability to confer a crucial effect on coral well-being under conditions that might lead to bleaching. *Stachybotrys chlorohalonata* exacerbated stress, whereas *C. halotolerans* conferred protective effects, reducing thermal stress impacts on coral physiology and bacterial communities. The need for functional dissection of the coral mycobiome within the complex mutual relationships with bacteria, algae, viruses, and host physiology within the holobiont is timely. Given the emerging understanding of the negative outcomes of mycobiome dysbiosis in other animals, along with the potential employment of fungal probiotics as prophylactics or therapeutic interventions [21], our results emphasize the potential of manipulating the coral mycobiome to mitigate stress in the presence of changing environments.

## Supplementary Material

Supplementary_third_revision_clean_wraf090

Supplementary_Table_S1_third_revision_wraf090

## Data Availability

Raw sequence data are available under NCBI BioProject PRJNA1198912. All fungal sequence accession numbers are provided in Supplementary Table S1.
