# Correction to: Beneficial and detrimental fungi within the culturable mycobiome of the Red Sea coral *Stylophora pistillata*

**DOI:** 10.1093/ismejo/wraf167

**Published:** 2025-09-04

This is a correction to: Lior Granit, Rotem Levi, Nofar Lifshitz, Guilhem Banc-Prandi, Einat Zelinger, Britt Ronen, Judith Kraut-Cohen, Ankur Naqib, Stefan J Green, Maoz Fine, Oded Yarden, Beneficial and detrimental fungi within the culturable mycobiome of the Red Sea coral *Stylophora pistillata, The ISME Journal*, Volume 19, Issue 1, January 2025, wraf090, https://doi.org/10.1093/ismejo/wraf090

A typographical error in the title of the originally published article is now emended to read: ``*pistillata*'' instead of: ``*pistilatta*''.

