# Supplementary material for: Beneficial and detrimental fungi within the culturable mycobiome of the Red Sea coral Stylophora pistillata
Source: ISME J. 2025 May 3;19(1):wraf090. doi: 10.1093/ismejo/wraf090 (PMC12090049; doi:10.1093/ismejo/wraf090)
Supplement: Supplementary_third_revision_clean_wraf090 [file supplementary_third_revision_clean_wraf090.pdf]

Supplementary Information for

Beneficial and detrimental fungi within the culturable mycobiome of the Red Sea coral *Stylophora pistilatta*

Lior Granit[1], Rotem Levi[1], Nofar Lifshitz[1], Guilhem Banc-Prandi[2], Einat Zelinger[3], Britt Ronen[4], Judith Kraut-Cohen[5], Ankur Naqib[6], Stefan J. Green[7], Maoz Fine[4] and Oded Yarden[1*]

[1]Department of Plant Pathology and Microbiology, The Robert H. Smith Faculty of Agriculture Food and Environment, The Hebrew University of Jerusalem, Rehovot, Israel & The Interuniversity Institute of Marine Sciences, Eilat, Israel.

[2]Laboratory for Biological Geochemistry, School of Architecture, Civil and Environmental Engineering, École Polytechnique Fédérale de Lausanne (EPFL), 1015, Lausanne, Switzerland

[3]Centre for Scientific Imaging, The Robert H. Smith Faculty of Agriculture, Food and Environment, The Hebrew University of Jerusalem, Rehovot, Israel.

[4]Department of Ecology, Evolution and Behavior, The Alexander Silberman Institute of Life Sciences, The Hebrew University of Jerusalem, Jerusalem, Israel & The Interuniversity Institute for Marine Sciences, Eilat, Israel

[5]Institute of Soil, Water and Environmental Sciences, Agricultural Research Organization, Volcani Center, Rishon LeZion 7505101, Israel

[6]Rush Research Bioinformatics Core Facility, Rush University, Chicago, IL, USA

[7]Genomics and Microbiome Core Facility, Rush University, Chicago, IL, USA

*To whom correspondence should be addressed: Oded.Yarden@mail.huji.ac.il

<u>List of contents:</u>

1. Supplementary Materials and Methods
2. Supplementary Tables
3. Supplementary Figures
4. References

1. Materials and Methods

Sampling of S. pistillata. Coral fragments (approx. 3cm in length) were collected, by SCUBA, from the reef adjacent to the Interuniversity Institute for Marine Sciences in Eilat (IUI), northern Red Sea (29.5_N, 34.9_E) from depths ranging 6-45m at different times points between 2021-2023. Fragments were withdrawn from their sterile zip-lock collection bags and processed shortly after collection at the IUI.

Isolation, culturing and identifying coral-derived fungi. Fragments were crushed under sterile conditions and spread on potato dextrose agar (PDA) plates supplemented with 500mg/L chloramphenicol. A second layer of PDA was applied over the fragments. Plates were incubated at 25°C for 3-28 days, and when fungal growth was visible, sections from the edge of the colony were repeatedly transferred to plates containing fresh medium until pure cultures were obtained. DNA was extracted from the cultures using the GenElute™ Plant Genomic DNA Miniprep Kit (Merck, Israel), or Invisorb® Spin Plant Mini Kit (INVITEK molecular, Germany) as described by the manufacturers. Initial molecular identification of fungi was based on amplification of DNA from the Internal Transcribed Spacer (ITS) region DNA (Table S2) as previously described [1-2]. PCR amplicons (~600 bp) were sequenced using the Sanger method at MCLAB laboratories (California, USA) and analyzed using SnapGene software (GSL Biotech). For *C. halotolerans* identification, partial actin gene sequences were selected for further sequence-based analysis ([3]; Table S2). *S. chlorohalonata* was identified at the Westerdijk Fungal Biodiversity Institute in The Netherlands. Genbank accession numbers of all amplicons obtained are provided in Table S1.

Relative abundance plot generation. Fungal population was assessed based on 198 ITS sequences. The bar plot was generated as a stacked bar chart using Python

(v3.11) and the matplotlib package [18]. A custom transformation function was applied to introduce a discontinuous x-axis, compressing values from 0–83% into the lower portion of the axis and expanding 83–100% above a visual break to enhance resolution of low-abundance taxa. Phylum-level annotations (Ascomycota, Basidiomycota, Mucoromycota) were indicated using bracket markers and labelled accordingly.

Fungal culturing conditions. *C. halotolerans* strain 0-36B-2a was cultured on PDA or in Potato Dextrose Broth (PDB, at 140rpm) at 28°C in the dark, and *S. chlorohalonata* strain was cultured on PDA at 28°C in the dark for 14 days. Conidia from the respective strains were harvested from 14-day old cultures (10 plates) by adding 8.5ml of cold, sterile, double distilled water (DDW) and detaching the conidia from the mycelium with sterilised glass beads. The conidial suspension was then filtered through a sterile funnel with a gauze pad and centrifuged at 4000g for 5 min at 4°C. The conidial pellet was washed three times with 30ml of cold sterile DDW. Conidial density was then calculated using a haemocytometer.

Coral inoculation with C. halotolerans and S. chlorohalonata conidia. A schematic representation of the experimental design used for coral inoculation experiments is presented in figure S1. Coral fragments (n=36) were collected from a single *S. pistillata* colony at 10m depth. Five fragments were subjected to PAM measurements and bacterial diversity assessment. The remaining 31 fragments were distributed across seven 1-L beakers containing seawater:three inoculated with *C. halotolerans*, two with *S. chlorohalonata*, both at a concentration of 10^6 conidia per ml while two served as controls. Beakers were shaken at 70rpm under 120µmol light at 25°C for 48 hours. To prevent an increase in salinity due to evaporation, the water

level in the beakers was monitored, and DDW was added, as needed, over the 48-hour period. Following rinsing, 20 fragments were transferred to the aquaria in the Red Sea Simulator system [4] at ambient (~25°C) or elevated (33°C) temperatures. The flow rate per aquarium was approximately 104 L per hour. Seawater was filtered through dual 120-mesh (130-micrometer) filters before entering four 2000 L mixing tanks. Wastewater was subjected to sand filtration before discharge into the sea [4]. Fragments were monitored for 96 hours before final sampling for PAM measurements and nucleic acid extraction.

Pulse Amplitude Modulated fluorometer (PAM) measurements. To evaluate the well-being of corals under thermal stress, the photochemical activity of their algal symbionts was measured through chlorophyll fluorescence using an Imaging-PAM (Walz GmbH, Germany) device. For each coral fragment, basal fluorescence (F_0) was determined by applying pulses of actinic light (~1 μmol photons m^{-2} s^{-1}). A subsequent saturating pulse (> 4000 μmol photons m^{-2} s^{-1}) was used to determine the maximal fluorescence (F_m). The variable fluorescence (F_v) was calculated as the difference between F_m and F_0. Measurements were taken after 20-30 minutes of dark acclimation to ensure that all PSII reaction centers were open, allowing for the measurement of maximum quantum yield. The maximum quantum yield of PSII was calculated using the F_v/F_m ratio.

Analysis of host stress-related gene expression. For gene expression analysis coral fragments were sampled at two time points: 0 hours and after 96 hours of incubation in the Red Sea Simulator aquaria, under the same experimental conditions described above (see Fig. S1). The analysis included fragments exposed to ambient

(25°C) or elevated (33°C) temperatures, inoculated with *C. halotolerans* or untreated (control). RNA was extracted from 3–4 biological replicates per treatment group (n=4 for Peroxiredoxin-6 and RAD51, n=3 for HSP70). Data were analyzed using two-way ANOVA followed by Tukey's HSD test to assess the effects of temperature and inoculation. RNA was extracted from crushed coral, which was ground into a fine powder with liquid nitrogen in a mortar and pestle, using TRIzol (Thermo Fisher Scientific, Israel) and 1-Bromo-3-chloropropane (BCP, Sigma Aldrich). The powdered fragments were mixed, by vortexing, in 15ml Falcon tubes with 1.5-2ml of cold TRIzol, followed by a 3-hour incubation on ice. The lysate was then transferred to a 2ml tube and centrifuged (12,000g at 4°C) for 10 minutes. The clear supernatant (~1.5 ml) was collected into a fresh tube. Cold BCP (150µl) was added, and the mixture was gently vortexed and incubated for 30 minutes at room temperature. Tubes were then centrifuged for 15 minutes after which the aqueous phase was transferred to a new tube. A second phase-separation was carried out by adding 500µl of TRIzol and 120µl of cold BCP, followed by incubation and centrifugation. Precipitated RNA was resuspended in 40µl of RNase-free water and incubated for 5 minutes at 55°C. The RNA was then treated with the TURBO DNA-free™ Kit (Invitrogen) to remove any contaminating DNA. The RNA was reverse transcribed using the High-Capacity cDNA Reverse Transcription Kit (Applied Biosystems, CA, USA) according to the manufacturer's instructions. Transcript abundance was measured using the Step One Plus Real-Time PCR system (Applied Biosystems, CA, USA) under standard conditions. cDNA samples were diluted to a concentration of 2 ng/µl and run in triplicate in 10µl qRT-PCR reactions, which included primers and SYBR Green Master Mix. Genes analyzed included Heat shock protein 70 (HSP70), peroxiredoxin-6 and Rad51 (Table S2). The comparative delta Ct (ΔCt) method was applied to analyze

the data, with fold changes calculated using the $2^{-\Delta Ct}$ formula to estimate relative transcript levels in each sample. All ΔCt values were normalized to the reference gene adenosyl-homocysteinase (AdoHcyase).

DNA extraction for assessment of bacterial communities. Coral samples were quickly transferred from the experimental site in sterile ziplock bags and stored at -80°C until further processed. For tissue recovery, each fragment was airbrushed in a sterile ziplock bag with sterile PBS. DNA was extracted from the tissue using the DNeasy PowerSoil Pro Kit (Qiagen). DNA samples were maintained at -20°C until transferred, using GenTegra-DNA tubes (GenTegra LLC, Pleasanton, California, USA). Microbial community structure analysis was performed using 16S rRNA gene amplicon sequencing with primers targeting the microbial V4 variable region [5], using a two-stage PCR protocol as described previously [6]. Library preparation was performed at the Genomics and Microbiome Core Facility (GMCF) of Rush University Medical Center, and sequencing on an Illumina NovaSeq6000 (2x258 base reads) was performed at the DNA Services facility at the University of Illinois at Urbana-Champaign.

Bioinformatics 16s rRNA amplicon analysis. Initial analyses of gDNA sequencing results were performed at GMCF as follows: Microbiome bioinformatics were performed with QIIME2 2023.5 [7]. Raw sequence data was checked for quality using FastQC and merged using PEAR. Merged sequences were quality filtered using the q2-demux plugin and denoised with DADA2 [8] (via q2-dada2). Primer adapter sequences were removed using the cutadapt algorithm. Taxonomy assignment was performed to generate ASVs using the q2-feature-classifier [9] classify-sklearn naïve

Bayes taxonomy classifier against the SILVA 138 99% reference sequences database. The contaminant removal software, decontam, was used to detect and remove any potential contaminants based on the prevalence of ASVs in four no-template controls (NTCs). These NTCs were prepared using reagent-grade water in place of DNA and were processed alongside the samples using the same mastermix during PCR. We expanded these analyses using Qiime2 (version 2024.5) within a WSL environment through Visual Studio Code. Sequence data were processed by initial filtering the samples to remove negative controls and samples with fewer than 1000 reads. To ensure the integrity of the dataset, an outlier detection analysis was performed in R (version 4.4.2) based on the Principal Coordinate Analysis (PCoA) using the Bray-Curtis distance matrix, resulting in a refined dataset consisting of 1,827 ASVs. These results, processed using the qiime2R package, included the primary coordinate axes (PC1 and PC2), which were used to represent the variation among samples. Metadata and PCoA results were merged to ensure consistency across samples. To evaluate variability within treatment groups and identify potential outliers, samples were grouped according to their respective experimental conditions. For each treatment group, the principal coordinates (PC1 and PC2) were extracted, and the centroid (mean position) of the group was calculated. The Mahalanobis [10] distance was then computed for each sample within a group to assess its multivariate distance from the group centroid. The covariance matrix of the group's principal coordinates was used to standardize the distances and account for the correlation between the variables. Distances were computed using standard statistical functions in R. Outlier detection was performed by defining a threshold of 0.7 standard deviations above the mean Mahalanobis distance for each group. Samples exceeding this threshold were classified as outliers. A total of 6 samples were identified as

outliers and were subsequently removed from downstream analyses. The refined dataset, which included 1,827 ASVs across 18 samples, was used for all subsequent diversity and taxonomic analyses. Taxonomic assignments were refined by filtering the feature table and representative sequences to remove mitochondria and chloroplast sequences, ensuring accurate downstream analyses. Repeated rarefaction (for 10,000 times) was performed to normalize sequencing depth, with a sampling depth of 1,000 reads. Taxonomic profiles were visualized as stacked bar plots using collapsed taxonomic tables at multiple hierarchical levels. Taxa representing less than 1% or ambiguous taxa were grouped and labelled as "other" (Figure S4). Alpha diversity was evaluated using Shannon diversity indices, with significant statistical group-wise comparisons performed via pairwise Kruskal-Wallis tests. Beta diversity was assessed using Bray-Curtis dissimilarity, and statistical significance across experimental groups was determined using PERMANOVA pairwise comparisons on the original matrix with 999 permutations. All analyses utilized experimental metadata to define groupings and statistical comparisons. Default parameters were applied unless specified otherwise.

Microscopy. Scanning Electron Microscopy (SEM) was performed using a JSM-IT100 microscope (JEOL). Coral fragments were first washed three times in filtered seawater (FSW) and then fixed for 12 hours in a 4% paraformaldehyde (PFA) solution at 4°C. After fixation, the fragments were washed three times for 10 minutes each in PBS and stored in PBS at 4°C for up to two weeks until further processing, as described by [11]. Dehydration was completed in 100% ethanol for 30 minutes before processing with a Critical Point Dryer (Quorum, K850). Finally, the dried fragments were coated with a 1nm layer of Au-Pd using a gentle coating at 12mA for 60 seconds

and subsequently examined with the SEM. Fragments intended for tissue sectioning and light microscopy were first fixed as described above and then subjected to skeleton de-calcification using a 20% (w/v) Ethylenediamine tetraacetic acid (EDTA) solution for 6-14 days, while changing the solution every 2-4 days. A second fixation process in PFA followed by three washes with PBS was then carried out. Prior to embedding of the tissue, fragments were stored overnight at 4°C in PBS containing 25% sucrose. Embedding was prepared in a Tissue-Plus Optimal Cutting Temperature (O.C.T) compound (Scigen) frozen with liquid nitrogen, and 20μm sections were cut using a cryostat to prepare slides of coral tissue to be examined using a light microscope.

2. Supplementary Tables

Table S1 Fungal taxa found in association with *Stylophora pistillata* coral (GeneBank accession numbers of SUB14854636 and SUB14841483), including two new taxa [12-13]. This table is provided as a separate file. <Supplementary_Table_S1.xlsx>.

Table S2 Summary of Primers used in this research.

Gene	Primers	Sequence (5'-3')	Amplicon size (bp)	Use	Source
**Internal Transcribed Spacer (ITS)**	ITS-5F	GGAAGTAAAAGTCGTAACAAGG	~600	Fungal strains identification	[14]
	ITS-4R	TCCTCCGCTTATTGATATGC			
**ACTINα(ACT)**	ACT-512F	ATGTGCAAGGCCGGTTTCGC	~270	Fungal strains identification	[3]
	ACT-783R	TACGAGTCCTTCTGGCCCAT			
**16Ss rRNA**	515F	ACACTGACGACATGGTTCTACAGTGTGYCAGCMGCCGCGGTAA	~320	Bacterial identification	Modified from [15] (underlined regions represent linker sequences)
	806R	TACGGTAGCAGAGACTTGGTCTGGACTACNVGGGTWTCTAAT			
**Adenosyl-Homocysteinase**	Sp_Adeno F2	CAGGCTGTCTGCACATGACAA		*S. pistillata* RT-PCR (RNA)	[16]
	Sp_Adeno R2	TCCCAATTCAGTCAATGTTTCAA			
**Heat Shock Protein 70 (Hsp70)**	Sp_hsp70_1F	AAGAATCCTCAGGTCCTAC			[17]
	Sp_hsp70_1R	GGCAACTCAAACAGGTAC			
**Peroxiredoxin-6**	Sp_pero_F	GGCAAGGACTGTATGGTTCTAC			
	Sp_pero_R	TTACCCGAAGGGCATTTCTC			
**Rad51**	Sp_rad_F	TTTGCTGGCCTCCAAAGTATAG			
	Sp_rad_R	TGTAACCAGGATGACGATGATG			

Table S3 Tests (Kruskal-Wallis) were performed to assess pair-wise differences in alpha diversity metrics between temperature and *C. halotolerans* inoculation regimes. P-values indicate the significance of observed differences; values below 0.05 are bolded and denote significant differences between groups.

Group 1	Group 2	p-value	q-value
Clad_25C_96hr (n=3)	Clad_33C_96hr (n=4)	1	1
Clad_25C_96hr (n=3)	Control_0hr (n=3)	**0.0495**	**0.1651**
Clad_25C_96hr (n=3)	Control_25C_96hr (n=4)	0.1573	0.2247
Clad_25C_96hr (n=3)	Control_33C_96hr (n=4)	0.1573	0.2247
Clad_33C_96hr (n=4)	Control_0hr (n=3)	0.1573	0.2247
Clad_33C_96hr (n=4)	Control_25C_96hr (n=4)	0.3865	0.4831
Clad_33C_96hr (n=4)	Control_33C_96hr (n=4)	0.1489	0.2247
Control_0hr (n=3)	Control_25C_96hr (n=4)	0.4795	0.5328
Control_0hr (n=3)	Control_33C_96hr (n=4)	**0.0339**	**0.1651**
Control_25C_96hr (n=4)	Control_33C_96hr (n=4)	**0.0209**	**0.1651**

Table S4 PERMANOVA (Permutational Multivariate Analysis of Variance) was conducted to evaluate differences in beta diversity across temperature and *C. halotolerans* inoculation regimes, based on Bray-Curtis dissimilarity matrices. Pairwise comparisons were performed to evaluate how fungal inoculation influenced microbial community composition. Each comparison relied on 999 permutations to calculate p-values, with significant differences identified when p<0/05.

Group 1	Group 2	Sample size	Permutations	p-value	q-value
Clad_25C_96hr	Clad_33C_96hr	7	999	**0.037**	0.062
Clad_25C_96hr	Control_0hr	6	999	0.196	0.218
Clad_25C_96hr	Control_25C_96hr	7	999	0.958	0.958
Clad_25C_96hr	Control_33C_96hr	7	999	**0.027**	0.062
Clad_33C_96hr	Control_0hr	7	999	**0.035**	0.062
Clad_33C_96hr	Control_25C_96hr	8	999	0.081	0.101
Clad_33C_96hr	Control_33C_96hr	8	999	**0.025**	0.062
Control_0hr	Control_25C_96hr	7	999	0.078	0.101
Control_0hr	Control_33C_96hr	7	999	**0.029**	0.062
Control_25C_96hr	Control_33C_96hr	8	999	**0.011**	0.062

3. Supplementary Figures

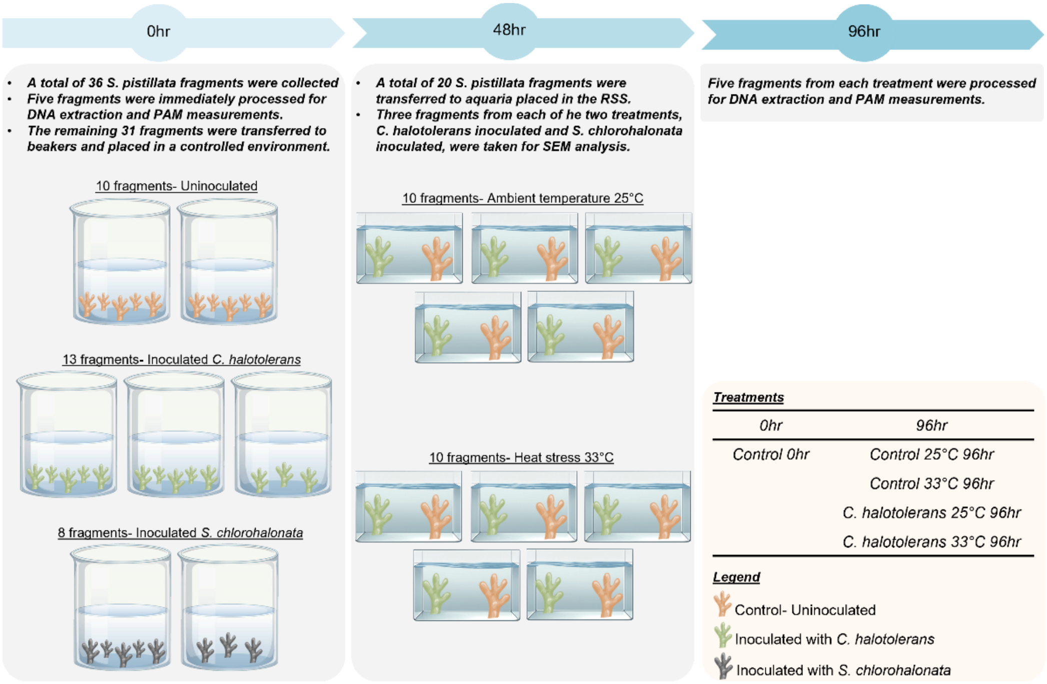

Figure S1. Experimental design for assessing the potential effect of *C. halotolerans* and *S. chlorohalonata* on *S. pistillata* well-being under ambient and heat stress condtions. The experiments were conducted at the IUI-Eilat using the Red Sea Simulator system. n=5.

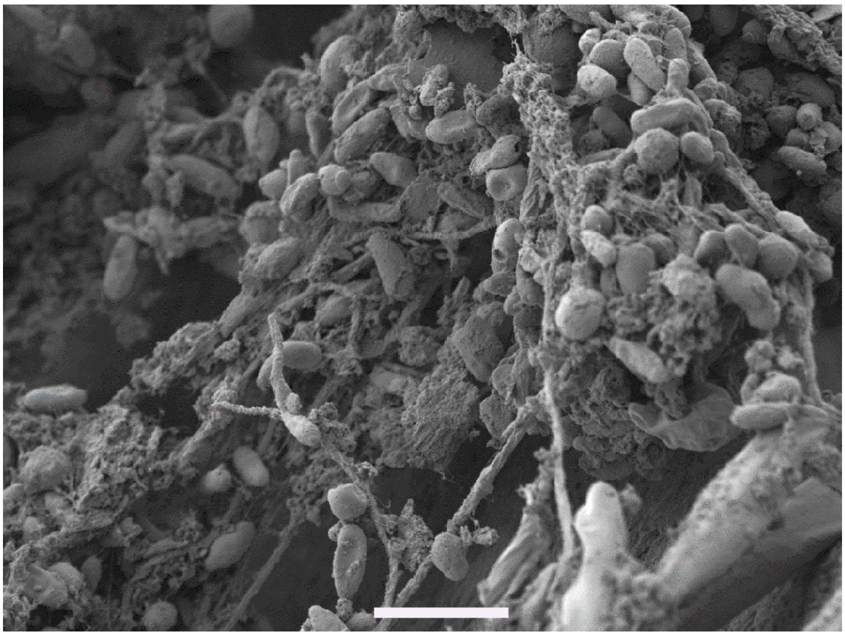

Figure S2. Scanning electron microscopy image showing *S. chlorohalonata* conidia adhering to the surface of deteriorating *S. pistillata* coral fragments after 48 hours of incubation. Image taken at 1900× magnification with an accelerating voltage of 2.00 kV using a JEOL SEM (scale bar = 10µm).

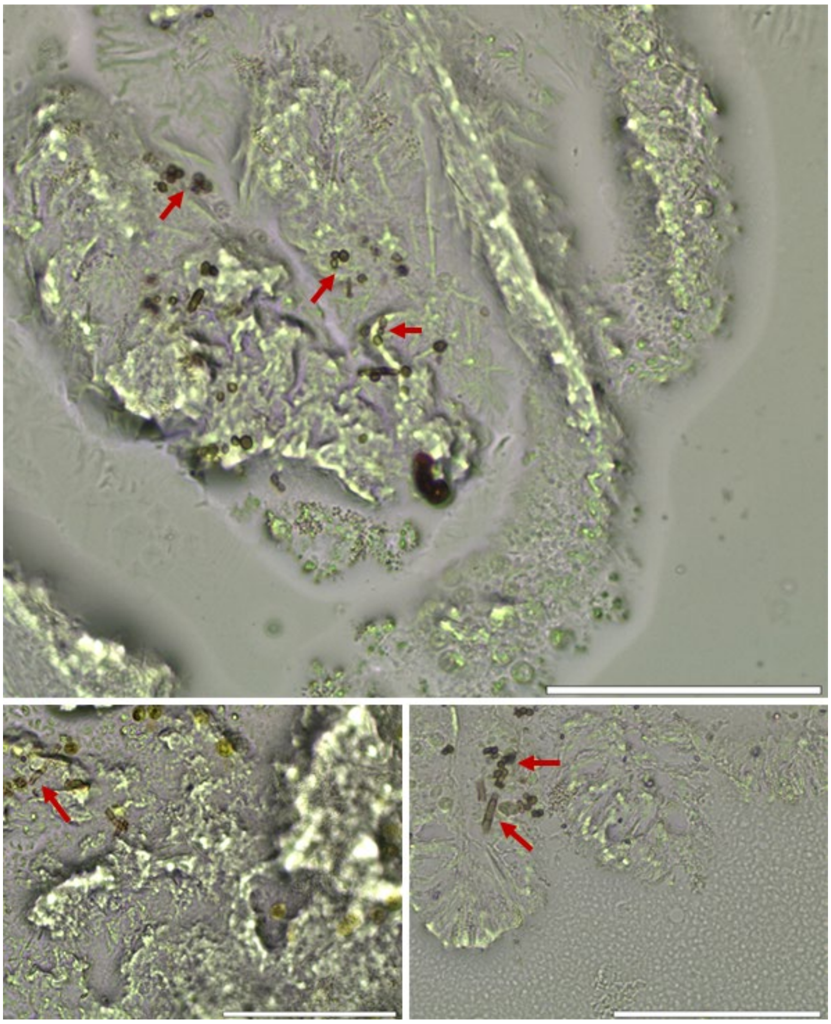

Figure S3. Bright-field microscope image showing conidia and hyphal fragments of *C. halotolerans* (marked by red arrows) in sections of decalcified *S. pistillata*.

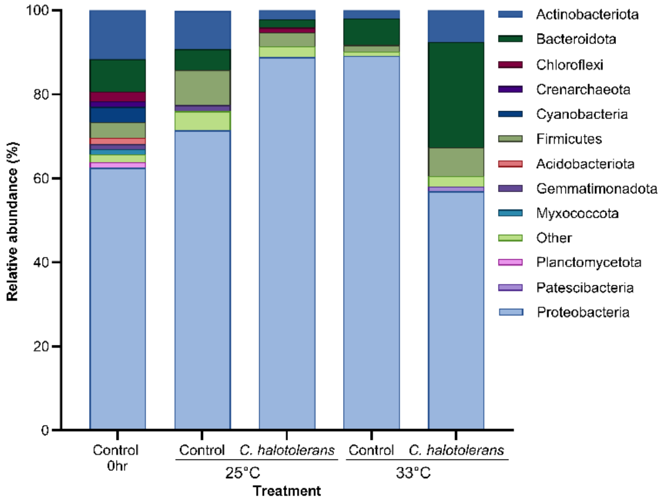

Figure S4. Relative abundance of the most dominant bacterial phyla in *S. pistillata* coral fragments as affected by inoculation with *C. halotolerans* and different temperatures, 96 hr after fungal inoculation. Only phyla with a relative abundance above 1% are displayed, with those below this threshold identified as "other". Results are based on n=3 or n=4 biological replicates per treatment.

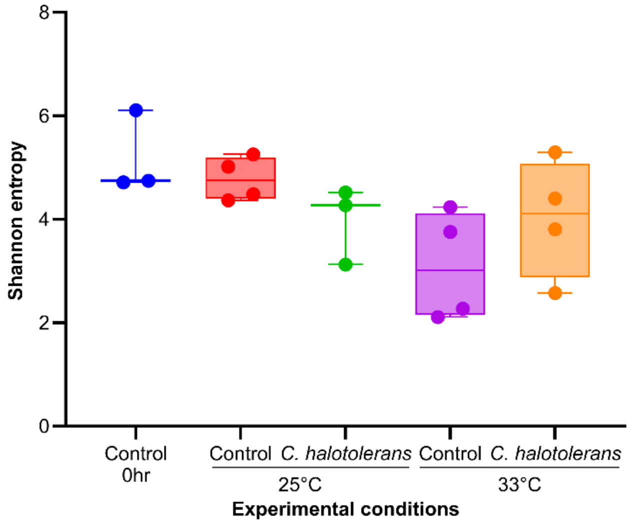

Figure S5 Shannon diversity index values of the microbial community 96 hr after fungal inoculation. Each boxplot represents a different experimental group illustrating the variation in alpha diversity under the specified temperature and *C. halotolerans* inoculation regimes. The statistical analysis is presented in Table S3.

4. References

1. Lifshitz N, Hazanov L, Fine M, Yarden O. Seasonal variations in the culturable mycobiome of *Acropora loripes* along a depth gradient. *Microorganisms*. 2020; 8(8):1139. https://doi.org/10.3390/microorganisms8081139

2. Schoch CL, Seifert KA, Huhndorf S, Robert V, Spouge JL, Levesque CA, Chen W. Fungal Barcoding Consortium. Nuclear ribosomal internal transcribed spacer (ITS) region as a universal DNA barcode marker for Fungi. *Proc Natl Acad Sci USA*. 2012; 109(16):6241-6246. https://doi.org/10.1073/pnas.1117018109

3. Carbone I, Kohn LM. A method for designing primer sets for speciation studies in filamentous ascomycetes. *Mycologia*. 1999; 91(3), 553–556. https://doi.org/10.1080/00275514.1999.12061051

4. Bellworthy J, Fine M. The Red Sea simulator: A high-precision climate change mesocosm with automated monitoring for the long-term study of coral reef organisms. *Limnol Oceanogr: Methods*. 2018; 16(6), 367–375. https://doi.org/10.1002/lom3.10250

5. Nguyen WQ, Chrisman LP, Enriquez GL, Hooper MJ, Griffin TL, Ahmad M, Rahman S, Green SJ, Seed PC, Guitart J, Burns MB, Zhou XA. Gut microbiota analyses of cutaneous T-cell lymphoma patients undergoing narrowband ultraviolet B therapy reveal alterations associated with disease treatment. *Front Immunol*. 2024; 14:1280205. https://doi.org/10.3389/fimmu.2023.1280205

6. Naqib A, Poggi S, Wang W, Hyde M, Kunstman K, Green SJ. Making and sequencing heavily multiplexed, high-throughput 16S ribosomal RNA gene amplicon libraries using a flexible, two-stage PCR protocol. *Methods Mol Biol*. 2018; 149-169. https://doi.org/10.1007/978-1-4939-7834-2_7

7. Bolyen E, Rideout JR, Dillon MR, Bokulich NA, Abnet CC, Al-Ghalith GA, Alexander H, Alm EJ, Arumugam M, Asnicar F, Bai Y. Reproducible, interactive, scalable and extensible microbiome data science using QIIME 2. *Nat Biotechnol*. 2019; 37(8), 852–857. https://doi.org/10.1038/s41587-019-0209-9

8. Callahan BJ, McMurdie PJ, Rosen MJ, Han AW, JohnsonAJA, Holmes SP. DADA2: High-resolution sample inference from Illumina amplicon data. *Nat Methods*. 2016; 13(7), 581–583. https://doi.org/10.1038/nmeth.3869

9. Bokulich NA, Kaehler BD, Rideout JR, Dillon M, Bolyen E, Knight R, Huttley GA, Gregory Caporaso J. Optimizing taxonomic classification of marker-gene amplicon sequences with QIIME 2's q2-feature-classifier plugin. *Microbiome*. 2018; 6, 1-7. https://doi.org/10.1186/s40168-018-0470-z

10. Cabana E, Lillo RE, Laniado H. Multivariate outlier detection based on a robust Mahalanobis distance with shrinkage estimators. *Stat Pap*. 2021; 62, 1583–1609. https://doi.org/10.1007/s00362-019-01148-1

11. Ziv C, Gorovits R, Yarden O. Carbon source affects PKA-dependent polarity of *Neurospora crassa* in a CRE-1-dependent and independent manner. *Fung Genet Biol.* 2008; 45(2), 103–116. https://doi.org/10.1016/j.fgb.2007.05.005

12. Crous PW, Boers J, Holdom D, Osieck ER, Steinrucken TV, Tan YP et al. Fungal planet description sheets. *Persoonia*. 2022; 48:261– 371. https://doi.org/10.3767/persoonia.2022.48.08

13. Crous PW, Osieck ER, Shivas RG, Tan YP, Bishop-Hurley SL, Esteve-Raventós F et al. Fungal planet description sheets. *Persoonia*. 2023; 50:158–310. https://doi.org/10.3767/persoonia.2023.50.05

14. White TJ, Bruns T, Lee SJ, Taylor J. Amplification and direct sequencing of fungal ribosomal RNA genes for phylogenetics. *PCR Protocols*. 1990; 18(1), 315–322. https://doi.org/10.1016/b978-0-12-372180-8.50042-1

15. Walters W, Hyde ER, Berg-Lyons D, Ackermann G, Humphrey G, Parada A, Gilbert JA, Jansson JK, Caporaso JG, Fuhrman JA, Apprill A. Improved bacterial 16S rRNA gene (V4 and V4-5) and fungal internal transcribed spacer marker gene primers for microbial community surveys. *mSystems*. 2016; 1(1), 10-1128. https://doi.org/10.1128/msystems.00009-15

16. Kvitt H, Rosenfeld H, Zandbank K, Tchernov D. Regulation of apoptotic pathways by *Stylophora pistillata* (Anthozoa, Pocilloporidae) to survive thermal stress and bleaching. *PLOS ONE*. 2011; 6(12), e28665. https://doi.org/10.1371/journal.pone.0028665

17. Maor-Landaw K, Levy O. Gene expression profiles during short-term heat stress; branching vs. Massive Scleractinian corals of the Red Sea. *PeerJ*. 2016; 4, e1814. https://doi.org/10.7717/peerj.1814

18. Hunter JD. Matplotlib: a 2D graphics environment, *Compu Sci Eng.* 2007; 9(03):90-95.10.1109/MCSE.2007.55
